# Complementing predictive coding

**DOI:** 10.3389/fpsyg.2012.00554

**Published:** 2012-12-18

**Authors:** Will Newsome

**Affiliations:** Department of Philosophy, Macquarie UniversitySydney, NSW, Australia

**A commentary on**

**Whatever next? Predictive brains, situated agents, and the future of cognitive science**

by Clark, A. (in press). Behav. Brain Sci.

Clark (in press) discusses virtues of the neurocomputational perspective of *predictive coding*^1^, as well as evidential, conceptual, and methodological limits. I expand on some such methodological limits by asking: (1) “Are processes of reducing informational surprise limited to biological brains?” and (2) “Can some hyperpriors be culturally contingent?” My goal is not to make any specific theoretical contribution, but rather to motion at what form a multi-level, multi-disciplinary approach to predictive coding should take.

Clark notes that predictive coding suggests a neurocentric perspective (21 ms). “But dig a little deeper,” he continues, and this model might extend to bodily and world-involving processes (Clark, in press). Clark has long defended a view of human cognition “extending” into “tools, notations, and media” (Clark, 1997, 2001, 2002, 2003, 2005, 2008; with Clark and Chalmers, 1998). Perhaps processes such as writing notes or asking your partner to remind you of your upcoming meeting could count as *extended* or *distributed* processes of reducing informational surprise. But what gains accrue from extending the scope of the predictive coding framework?

First, let us consider how extending the framework is to pan out. One way of carving up philosophical perspectives on extended cognition is into two “waves” (Sutton, 2010). These waves map onto possible interpretations of extended predictive coding systems. The first wave is based on functional *parity* between the inner and the outer (Clark and Chalmers, 1998): if some process involving external and internal resources is functionally equivalent to an inner, recognizably cognitive process, then the hybrid process is *cognitive* as well. Similar reasoning could underlie inferences to extended predictive coding systems if some hybrid system could realize the process of error prediction, but I have trouble imagining a possible example without employing extreme science fiction. Let's consider an Alzheimer's patient, Otto, and his trusty notebook filled with his memories and useful information (from Clark and Chalmers, 1998). Are there clear hierarchical levels with corresponding processing functions [e.g., top-down (predictive), bottom-up (sensory), and error calculating] in the hybrid Otto-notebook system? Do we have here an error-reducing system (i.e., Otto's relevant neural circuitry) within an error-reducing system (i.e., the hybrid Otto-notebook system)? The worry arises that predictive coding is being overstretched. A more tenable extension lies with the second wave. In contrast to *parity*, second-wave extended cognition takes the role of external artifacts and media to be different from but *complementary* to that of brains (Sutton, 2010). In some “techno-dream” cases, external media (interacting with internal resources) will realize predictive processes in an analogous fashion to neural activity (*parity*), but, in most realistic cases (like Otto and his notebook), will *complement* the neural processes of reducing informational surprise by fulfilling different functional roles than the neural circuitry. Extending the predictive coding framework by *complementarity* thus necessitates a “distribution of explanatory weight” between the predictive coding framework and “approaches that explore or uncover the more idiosyncratic or evolutionary path-dependent features of the human mind, and the complex transformative effects of the socio-cultural niche” (Clark, 28 ms). It seems Clark follows this second-wave perspective in stating that predictive coding models of neural functioning could help make sense of the “complementary roles of morphology, action, and environmental structuring.” (22 ms) Thus, human cognitive architecture is more complex than the brain and predictive coding is only part of the “cognitive story.” As Clark shows, the process of minimizing informational surprise certainly goes beyond the brain—as action selectively samples the environment to maximize the likelihood of prior predictions—but is complemented and potentially made ever more complex by environmental scaffolding.

The second question concerning the possibility of cultural hyperpriors addresses similar methodological limits of predictive coding. Hyperpriors are introduced in Clark's discussion of binocular rivalry. Binocular rivalry occurs when a subject is presented two different images, one in each eye. Subjects report a slow switching between one percept and the other. Why the images do not combine in a single percept is explained, within the predictive coding view, by the fact that the system has the “general knowledge (…) that, for example, houses and faces are not present in the same place, at the same scale, at the same time.” (Clark, 6 ms). This knowledge is a hyperprior: a very general fact about embodied interaction with the physical world that structures and constrains prior predictions and how they interact. If it weren't for this general feature, there wouldn't necessarily be competition between the different images in the case of binocular rivalry.

How general must some knowledge be in order to count as a hyperprior? Are there more and less general kinds of knowledge that structure and constrain prior predictions? There is likely general *physical* knowledge such as Clark describes, but perhaps also general *cultural* knowledge concerning things like normal standing distance between interlocutors of certain kinds. Cultural hyperpriors would likely need to be described by the “path-dependent disciplines” rather than the predictive coding framework alone, thus further necessitating the multi-level, multi-disciplinary methodology seemingly required for investigating *specific real-world* processes of reducing informational surprise. As Clark puts it,

“this again raises questions about the eventual spread of explanatory weight between the framework on offer and any additional resources that may be required to determine the nature and shape of the hyperpriors that shape the processing and experience of evolved life-forms.” (25 ms).

In conclusion, the potential practical value of the predictive coding framework in complementing and cooperating with the disciplines studying path-dependent phenomena is yet to be determined. However, I venture the bet that, despite predictive coding's success in constructing models and describing neurocomputational activity, even larger strides will be made through such a multi-level, multi-disciplinary approach.

## Footnotes

^1^Briefly, the idea that brains are in the business of reducing informational surprise (i.e., the difference between prior predictions and sensory input) by changing prior predictions or by changing sensory input (via selective sampling or movement).
